# Left Amygdala Regulates the Cerebral Reading Network During Fast Emotion Word Processing

**DOI:** 10.3389/fpsyg.2020.00001

**Published:** 2020-01-23

**Authors:** Kimihiro Nakamura, Tomoe Inomata, Akira Uno

**Affiliations:** ^1^Section of Systems Neuroscience, National Rehabilitation Center Research Institute, Tokorozawa, Japan; ^2^Faculty of Human Sciences, University of Tsukuba, Tsukuba, Japan

**Keywords:** emotion words, reading, affective priming, amygdala, functional connectivity, repetition suppression and enhancement

## Abstract

Emotion words constitute a special class of verbal stimuli which can quickly activate the limbic system outside the left-hemisphere language network. Such fast response to emotion words may arise independently of the left occipitotemporal area involved in visual word-form analysis and rely on a distinct amygdala-dependent emotion circuit involved in fearful face processing. Using a hemifield priming paradigm with fMRI, we explored how the left and right amygdala systems interact with the reading network during emotion word processing. On each trial, participants viewed a centrally presented target which was preceded by a masked prime flashed either to the left or right visual field. Primes and targets, each denoting negative or positive nouns, could be either affectively congruent or incongruent with each other. We observed that affective congruency produced parallel changes in neural priming between the left frontal and parietotemporal regions and the bilateral amygdala. However, we also found that the left, but not right, amygdala exhibited significant change in functional connectivity with the neural components of reading as a function of affective congruency. Collectively, these results suggest that emotion words activate the bilateral amygdala during early stages of emotion word processing, whereas only the left amygdala exerts a long-distance regulatory influence over the reading network *via* its strong within-hemisphere connectivity.

## Introduction

Fluent reading begins with fast visual analysis of written words which in turn activates multiple neurocognitive systems involved in language processing. Cognitive models of reading generally assume that fine-grained visual analysis of letter-strings is a pivotal step preceding phonological activation, meaning comprehension and spoken production (Seidenberg and McClelland, 1989; Coltheart et al., 2001). At the neural level, this initial orthographic processing relies on the left occipitotemporal region associated with higher order visual recognition (Dehaene et al., 2005; Thesen et al., 2012). In parallel with the cognitive models of reading, the occipitotemporal region is known to have structural and functional coupling with other left-hemisphere regions, including the lateral temporal area associated with meaning (Dehaene and Naccache, 2006), inferior parietal area involved in phonological conversion (Price, 2012), and frontotemporal junction area involved in pronunciation (Klein et al., 2015; Stevens et al., 2017). These neural structures form a mature reading network across variously different writing systems (Bolger et al., 2005; Nakamura et al., 2012a). Interestingly, however, recent neuroimaging data suggest that skilled reading involves a broader set of neural structures than thought previously, including the sensorimotor and limbic systems. For example, the sensorimotor cortex outside the classical reading network is shown to play a role in semantic processing of nouns and verbs associated with body parts and their actions (Willems et al., 2010; Grisoni et al., 2016; Mollo et al., 2016).

Likewise, the efficient reading network may extend to neural emotion circuits involved in prosody and fear processing. For example, recent observations suggest that emotional prosodic sensitivity plays a greater role in normal reading development than known previously (Goswami et al., 2016; Suarez-Coalla et al., 2016; Kocaarslan, 2019). In fact, emotional words constitute a special class of verbal stimuli which quickly activate the evolutionarily older limbic system, including the amygdala and cingulate cortex in both hemispheres, well outside the typical reading network (see Citron, 2012 for review). In particular, the amygdala complex is known to rapidly respond to emotion words at an early stage of reading, i.e., ~200 ms after stimulus onset (Naccache et al., 2005; Gaillard et al., 2006; Ponz et al., 2014), which is almost identical to the known response latency of the occipitotemporal visual word-form area (VWFA) responsible for orthographic processing (Cohen et al., 2000). This in turn suggests that the amygdala can detect the emotional content of written words even before orthographic and subsequent stages of word processing (e.g., phonological and lexico-semantic) occur in the left-hemisphere network. Indeed, such early sensitivity to emotional content may arise from neural systems outside the classical reading network, in particular, a direct pathway linking the amygdala with early visual regions and subcortical structures, which is shown to be functioning during the fast recognition of fearful faces (Noesselt et al., 2005; Gschwind et al., 2012; Burra et al., 2019). This sounds plausible given the fact that we can easily read affective meanings from character-like emoticons (e.g., 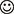 and 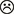) and seems to concur with the notion that cultural acquisitions, such as reading and arithmetic, rely on the “neuronal recycling” of pre-existing brain circuits (Dehaene and Cohen, 2007).

In fact, a functional magnetic resonance imaging (fMRI) study by Tabert et al. (2001) showed strong functional coupling between the extrastriate cortex and the amygdala in the right hemisphere during emotion word processing. More recently, however, Herbert et al. (2009) observed functional connectivity between the extrastriate cortex and amygdala in the left hemisphere during reading of affective adjectives. These observations therefore support the putative contribution of the extrastriate-amygdala route to the fast processing of emotion words, but seem conflicting in terms of the hemispheric dominance in functional connectivity. More generally, the existing neuroimaging literature of reading seems rather inconsistent as to how the left- and right-hemisphere systems contribute to affective word processing, since activation patterns of the amygdala and extrastriate cortex have been reported as being “left-lateralized” (Hamann and Mao, 2002; Kensinger and Schacter, 2006; Lewis et al., 2007), “right-lateralized” (Van Strien and Heijt, 1995; Tabert et al., 2001; Rochas et al., 2014), or “bilateral” (Eviatar and Zaidel, 1991; Naccache et al., 2005; Nakic et al., 2006).

On the one hand, it is possible that the left-hemisphere system, including the amygdala, plays a primary role in emotion word processing, because reading *per se* is a cultural skill relying on the left-hemisphere language network (Dehaene and Naccache, 2006; Price, 2012). On the other hand, it is also possible that the right-hemisphere system plays a general role in early emotion processing, not only for non-verbal stimuli (e.g., faces, animals and visual scenes) (Noesselt et al., 2005; Mormann et al., 2011; Gainotti, 2012; Bruder et al., 2017) but also for written words. Indeed, the right hemisphere is shown to be more efficient in extracting coarse semantic information from written words (Beeman et al., 1994) and thus can be more sensitive to basic categories of affect (e.g., pleasantness, fear, threat) than the left hemisphere even during reading. However, another interesting possibility is that the left- and right-hemisphere systems may contribute to emotion word processing differently from each other (Landis, 2006; Abbassi et al., 2011). In particular, it has been proposed that the left amygdala first detects the affective meaning of written words and then modulates the cortical activity involved in subsequent stages of word processing (Landis, 2006). In contrast, the right amygdala may well be equally sensitive to affective meanings during early word processing as described above, but may exert no or only weak modulatory influence over the reading network because of non-efficient callosal transfer. Accordingly, we hypothesized that emotion words activate the bilateral amygdala in early stages of visual word processing, whereas only the left amygdala interacts with the cerebral language network *via* its strong structural-functional connectivity within the left hemisphere.

**Figure 1:**
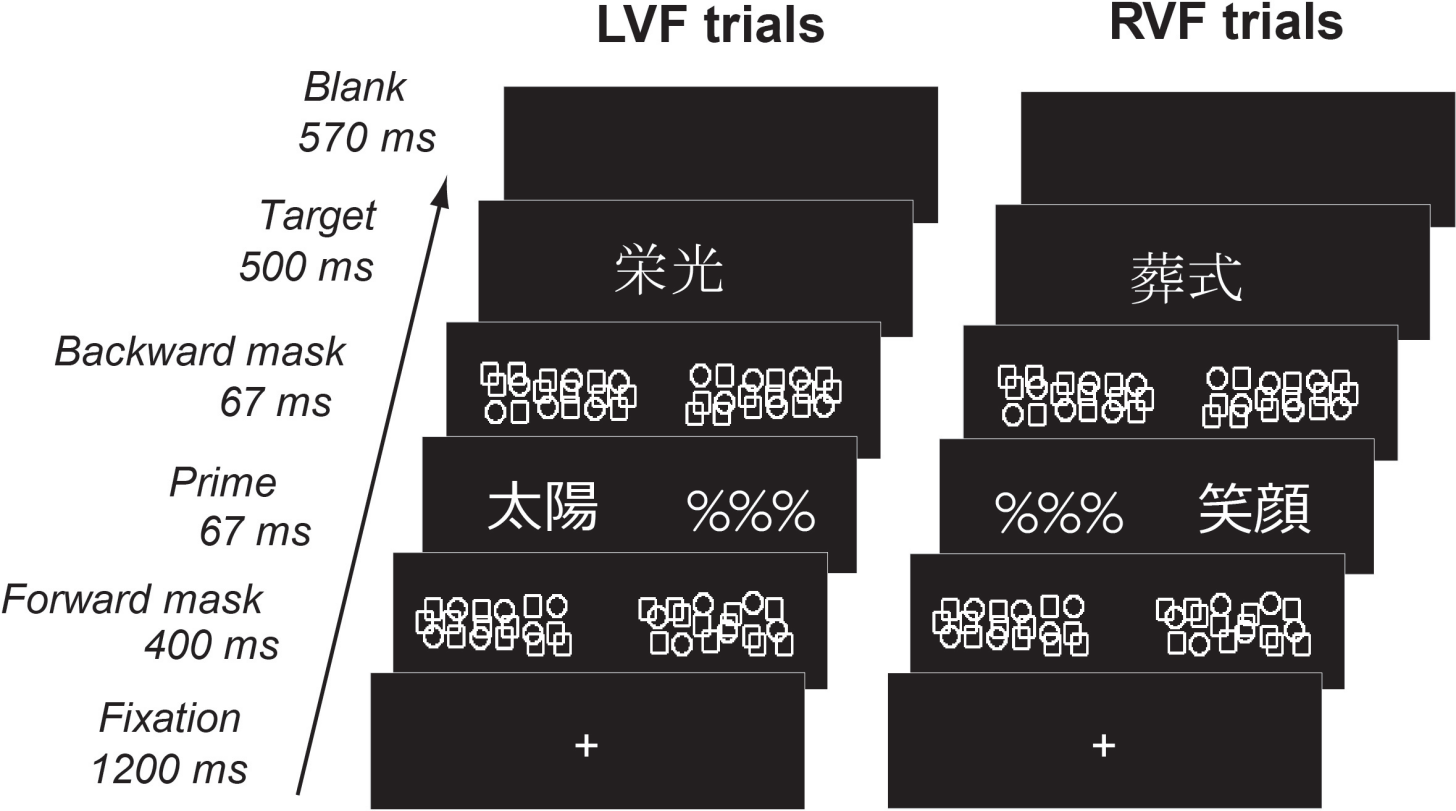
Behavioral paradigm. Each trial consisted of a masked prime flashed to LVF or RVF and a visible target displayed on the center of the screen. Primes and targets, each representing either positive or negative nouns, could be either congruent or incongruent with each other in emotional valence. Participants determined whether visible targets represented concrete objects or abstract concepts.

In the present study, we used a hemifield priming paradigm (Figure 1) with fMRI. On each trial, participants made semantic judgment about a centrally presented target, which was preceded by a masked prime flashed either to the left or right visual field (LVF or RVF). By manipulating the affective congruency between primes and targets (congruent or incongruent in emotional valence), we maximized the likelihood for isolating neural systems sensitive to the emotional valence of written words and their hemispheric bias. Several past studies used similar hemifield paradigms to explore hemispheric lateralization during affective processing (Williams and Mattingley, 2004; Noesselt et al., 2005; Landis, 2006; Rochas et al., 2014). By delivering masked prime stimuli briefly to each hemifield, this priming paradigm enabled us to stimulate each hemispheric system separately during the early, automatic stage of word processing, since weak neural activation induced by such degraded primes occurs locally within each hemisphere and does not spread across hemispheres (Reynvoet and Ratinckx, 2004; Pas et al., 2016). We further performed functional connectivity analysis to assess long-distance interactions between the amygdala and the left-hemisphere reading network.

## Methods

### Participants

Fifteen healthy undergraduate students (five females, age range 20–22 years) participated in the present study. All participants were right-handed native speakers of Japanese and gave written informed consent prior to the experiments. Two participants were excluded from behavioral and brain imaging analyses because of low accuracy level (< 85%) or excessive head motion (> 2.5 mm). The protocol of this study was approved by the ethics committee of Kyoto University Graduate School of Medicine.

### Stimuli and Task

We selected 96 Japanese nouns written with two logographic characters (kanji) for visual stimuli. Half of them had emotionally positive meanings (e.g., smile, sun, success, love) whereas the other half negative meanings (e.g., funeral, war, despair, decline). In the positive set of words, half represented concrete objects (e.g., smile, sun), whereas the other half abstract concepts (e.g., success, love). Likewise, half of the negative set represented concrete objects (e.g., funeral, war) and the other half abstract concepts (e.g., despair, decline). Therefore, the stimulus set consisted of four groups of 24 words, each having (1) emotional valence either positive or negative and (2) concreteness either concrete or abstract. Word frequency was matched both between positive and negative words (8.26 vs. 7.92 in log frequency of occurrence) and between concrete and abstract words (8.00 vs. 8.19) according to the NTT Psycholinguistic database (Amano and Kondo, 2000). To verify the emotional valence and concreteness of the 96 words, we asked 56 healthy volunteers to rate each of the items with a 7-point scale (emotional valence: 1 = very negative to 7 = very positive, concreteness: 1 = very abstract to 7 = very concrete). Mean emotional valence and concreteness of each set are summarized in Table 1.

**Table 1:** Emotional valence and concreteness for each set of words [Mean (SD)]. Chi-square tests confirmed that emotional valence and concreteness were not affected by each other (*p* > 0.8 for both).

	Concrete	Abstract
**Emotional valence**		
Negative	2.25 (0.43)	2.43 (0.33)
Positive	4.82 (0.49)	5.48 (0.44)
**Concreteness**		
Negative	5.41 (0.52)	3.31 (0.44)
Positive	6.31 (0.37)	3.28 (0.46)

Each trial consisted of central fixation, a forward mask (~3.0° in visual length), a masked prime and a visual foil (“%%%,” ~3.0°), a backward mask (~3.0°) and a centrally-presented target (3.0°) (Figure 1). Each target was followed by a blank period for ~570 ms such that the stimulus-onset-asynchrony for targets was set to 2.8 s without jittering. The forward and backward masks were created by semi-random arrangement of circle and square shapes with the same line thickness as character fonts and centered on the left and right sides (3.0°) of the central fixation cross. Masked primes and visual foils were assigned pseudo-randomly either to LVF or RVF with a probability of 50%. Primes and targets were either emotionally congruent (i.e., same in valence) or incongruent (i.e., different in valence) with each other. On the dimension of concreteness, primes and targets were always congruent with each other, whereby concrete targets and abstract targets were preceded by concrete primes and abstract primes, respectively. Therefore, while the emotional valence of stimuli might be rather unevenly polarized (see Table 1), the effect of affective congruency priming was kept orthogonal to the functional requirements of the behavioral task.

In general, masked primes presented for >50 ms are partially visible for most healthy participants (Kouider et al., 2007), but we used the present experimental setting because (1) reliable effects of behavioral and fMRI priming can be obtained with masked words in parafoveal vision (Nakamura et al., 2012b; Pas et al., 2016) and (2) the present level of prime duration still allows assessing early and automatic stages of reading (Greenwald et al., 1996; Qiao et al., 2010; Nakamura et al., 2012a). On each trial, participants decided as quickly and accurately as possible whether targets represented concrete objects or abstract concepts by pressing keys with their left and right thumbs. Each participant received four sessions, each consisting of 240 trials (24 trials per condition and 48 word-absent baseline trials per session).

### Functional Magnetic Resonance Imaging Procedures

Imaging data were acquired in Kyoto University Hospital using a Siemens Trio 3 Tesla head scanner with a standard head coil optimized for a gradient echo-echo planar imaging (22 contiguous axial slices, thickness 4 mm with 1 mm gap, TR = 1,400 ms, TE = 30 ms, flip angle = 90°, field-of-view = 256 × 256 mm^2^, 64 × 64 pixels). High-resolution T1 anatomical images were obtained after the main experiment (160 contiguous axial slices, thickness 1 mm without gap, TR = 2,000 ms, TE = 3.39 ms, inversion time = 990 ms, flip angle = 8°, field-of-view = 176 × 192 mm^2^, 176 × 192 pixels). Each participant received four scanning sessions, each lasting 700 s and yielding 500 volumes.

### Data Analysis

Functional imaging data were analyzed using SPM12^1^. Images from each subject were corrected for head movements, normalized to the MNI template with a 2 × 2 × 2 mm^3^ voxel size, and spatially smoothed with an isotropic Gaussian filter (5 mm width at half maximum). These images were high-pass filtered at 120 s and smoothed with a 4 s Gaussian kernel. For each participant, a weighted-mean image for each contrast was computed by fitting each voxel time-series with the known time-series of the eight event types convolved with a canonical hemodynamic response function and its temporal and dispersion derivatives. These contrast images were submitted to the second-level analysis using analysis of variance (ANOVA). Response suppression and enhancement were calculated as decrease and increase of activation in congruent trials (i.e., primes and targets share the same valence) relative to incongruent trials, respectively (Henson and Rugg, 2003). In particular, crossover interaction between prime valence and affective priming was calculated as response suppression for positive primes and response enhancement for negative primes and response irrespective of hemifield. Unless stated otherwise, statistical significance was assessed with voxel-level *p* < 0.001 and cluster-level *p* < 0.05 corrected for multiple comparisons with family-wise error. To examine priming effects in the amygdala more closely, we used two 5-mm radius spherical regions-of-interest (ROIs) each centered at the left basolateral amygdala (−30, −4, −34) and its right homologous site (30, −4, −34) previously associated with masked emotion words (Naccache et al., 2005) and fearful faces (Noesselt et al., 2005). For each ROI, neural effects of affective congruency (“affective priming”) were assessed for each priming condition with voxel-level *p* < 0.05, corrected for multiple comparisons.

We next performed psychophysiological interaction (PPI) analyses (Friston et al., 1997) to assess functional connectivity with amygdala during emotion word processing. In brief, PPI computes functional coupling between a seed ROI and all other regions induced by psychological context. Since the whole-brain SPM revealed robust changes in neural priming as a function of prime valence (see section “Results”), we selected this valence-by-priming interaction as a critical contrast for assessing fast changes in functional connectivity during emotion word processing. Regional responses per session per participant were extracted by calculating the principal eigenvariate across all voxel for each of the left and right amygdala ROIs described above. For each participant, the PPI regressor was calculated as an element-by-element product of the amygdala response (physiological regressor) and a vector coding for the valence x priming interaction (psychological regressor) for each ROI. A whole-brain general linear model was computed using the three types of regressors for each participant. Contrast images representing the PPI were created for each ROI for each participant and submitted to the ANOVA treating the side of the amygdala (left vs. right) as a within-participant factor. Since the whole-brain SPM identified the left inferior frontal gyrus (IFG), left anterior cingulate gyrus (ACG), left posterior parietal cortex (PPC), and left occipitotemporal cortex (OTC) as neural correlates of the valence x priming interaction (see “Results”), we examined functional connection strength with the amygdala in this left-hemisphere network involved in emotion word processing (*p* < 0.05 corrected for multiple comparisons).

Additionally, we performed *post hoc* power analysis for ROI analyses using G*Power^2^. The *post hoc* assessment of statistical power is crucial for the present ROI analysis whose condition effects were smaller compared to those obtained in the highly conservative whole-brain analysis. We calculated the effect-size threshold $\eta_{p}^{2}$ by converting the voxel-level *Z* = 1.67 (corresponding to uncorrected *p =* 0.05) with the sample size of 13 participants. The obtained effect-size ($\eta_{p}^{2}$ = 0.21) was submitted to *post hoc* power analysis for within-participant factor repeated measures ANOVA with the following parameters: *α* = 0.05, number of measures = 8, non-sphericity correction *ε* = 1. We confirmed that the actual power of the experiment was sufficiently high (93.53%) despite the limited sample size. This can be attributed to the well-known power advantage of repeated measures designs which reduces the amount of error variance by factoring out the error term derived from between-participant variability (Keselman et al., 2001; Morgan and Case, 2013).

## Results

### Behavioral Results

**Figure 2:**
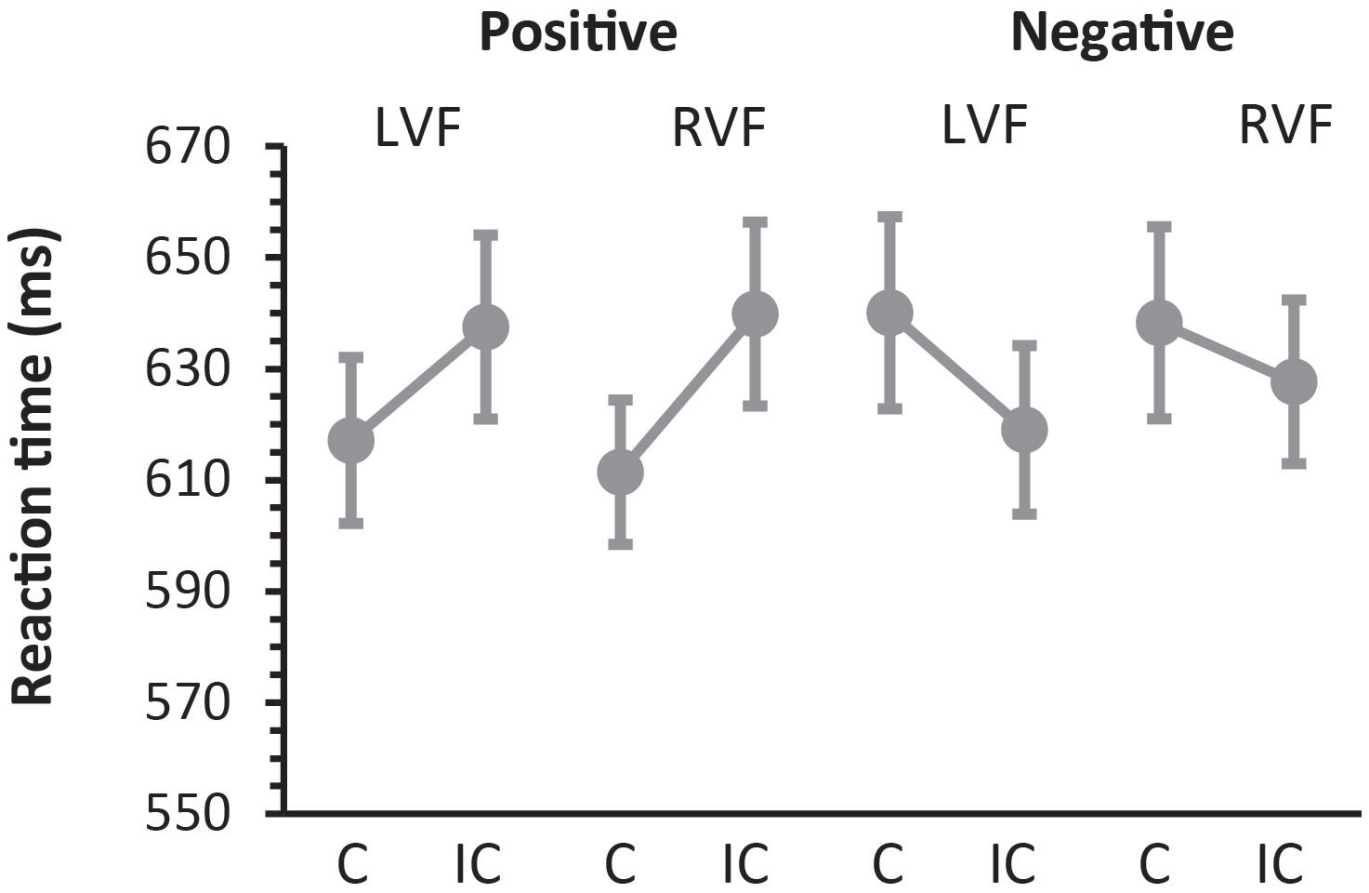
Behavioral results. Median reaction times (SEM) during the concrete/abstract judgment task as a function of prime valence (positive and negative) and prime hemifield (LVF and RVF). Participants responded faster to congruent (C) targets than to incongruent (IC) targets when masked primes were positive in valence. In contrast, participants responded faster to incongruent targets than to congruent targets when masked primes were negative in valance. This reversal of priming directions was observed on both LVF and RVF trials and confirmed by a robust cross-over interaction between the effects of valence and affective priming (see section “Results”).

Mean accuracy (SD) during the concrete/abstract judgment task was 89.95 (4.25) %. Median reaction times for correct responses are presented in Figure 2. We first submitted the RT data to Kolmogorov–Smirnov test for normality and Levene test for homoscedasticity and confirmed that these RT data met the assumption of normality (*p* = 0.15) and homogeneity of variance (*p* > 0.5) required for subsequent ANOVAs. We then performed a 2 × 2 × 2 ANOVA treating the effects of prime valence (positive vs. negative), prime hemifield (LVF vs. RVF) and affective priming (congruent vs. incongruent), as within-participant factors. The main effects of valence and priming were both non-significant [*F*(1, 12) = 3.43, *p* = 0.089, $\eta_{p}^{2}$ = 0.22 and *F*(1, 12) = 3.40, *p* = 0.90, $\eta_{p}^{2}$ = 0.22, respectively]. However, these effects showed a robust cross-over interaction with each other [*F*(1, 12) = 35.50, *p* < 0.0001, $\eta_{p}^{2}$ = 0.74], suggesting that the directions of affective priming changed with valence. The effect of hemifield never approached the level of significance (*F* < 1) but interacted with the effect of priming, suggesting that the overall magnitude of priming was greater for RVF than for LVF [11 vs. 0 ms, *F*(1, 12) = 5.14, *p* = 0.043, $\eta_{p}^{2}$ = 0.30, see below for further analyses]. Other interactions were all non-significant (*p* > 0.2 for all).

To further assess the observed interaction between valence and congruency, we then examined the effects of affective priming separately for negative and positive primes. When the analysis was restricted to positive primes, participants responded faster on congruent trials than on incongruent trials (614 vs. 639 ms), yielding a significant facilitatory effect of affective priming [*F*(1, 12) = 31.72, *p* = 0.0001, $\eta_{p}^{2}$ = 0.73]. This facilitatory priming did not interact with the effect of hemifield, suggesting that the magnitude of priming did not differ between LVF and RVF [*F*(1, 12) =2.98, *p* = 0.11, $\eta_{p}^{2}$ = 0.20]. On the other hand, when restricted to negative prime trials, participants responded more slowly on congruent trials than on incongruent trials (639 vs. 623 ms), thus yielding a significant inhibitory effect of priming [*F*(1, 12) = 16.89, *p* = 0.0015, $\eta_{p}^{2}$ = 0.59]. The magnitude of the inhibitory priming did not differ between LVF and RVF [*F*(1, 12) = 2.48, *p* = 0.14, $\eta_{p}^{2}$ = 0.17]. To summarize, these findings show that masked primes produced different patterns of affective priming as a function of valence, whereas these effects did not change with the prime hemifield.

### Imaging Results

**Figure 3:**
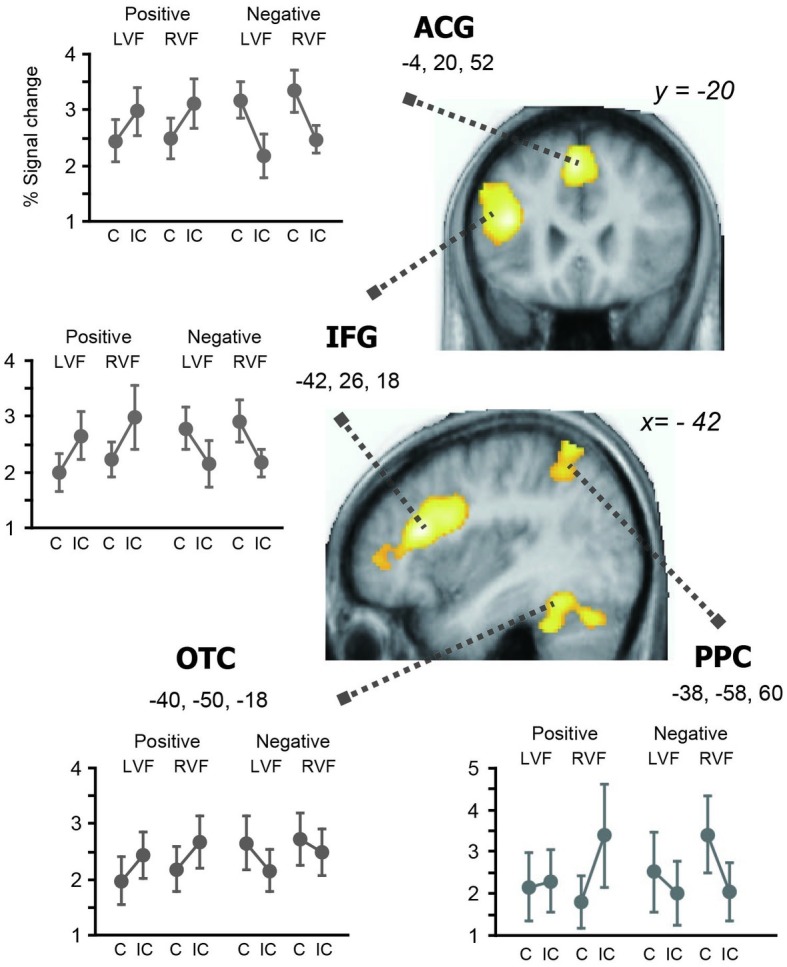
Brain regions showing cross-over interaction between valence and affective priming. For each region, percent signal change relative to the baseline is plotted against the valence (positive and negative) and prime hemifield (LVF and RVF). The whole-brain SPM analysis identified three left hemisphere regions showing significant changes in priming directions associated with the reversal of behavioral priming. That is, the left IFG, ACC, PPC, and OTC showed repetition enhancement associated with inhibitory priming (i.e., to negative primes) and repetition suppression associated with facilitatory priming (i.e., to positive primes), respectively.

The concrete/abstract semantic judgment task broadly activated the bilateral frontoparietal and temporal regions relative to the baseline. We assessed the effects of prime valence, prime hemifield, and affective congruency priming and their interactions. In parallel with the behavioral data, the main effects of affective priming and valence were both non-significant, but showed robust interaction in the left hemisphere (Table 2 and Figure 3). In particular, we observed a large cluster in the left IFG which included the local maximum at the ventrolateral part (−42, 26, 18, *Z* = 5.33) and two subpeaks in the orbital part (−48, 44, 4, *Z* = 5.10; −48, 36, 10, *Z* = 4.82). The same valence-by-priming interaction was also found in the left OTC (−40, −50, −18, *Z* = 4.56), bilateral ACG (−4, 20, 52, *Z* = 5.17), and left PPC (−38, −58, 66, *Z* = 4.30). Indeed, activation profiles of these regions revealed that the directions of neural priming changed with the affective valence of primes, i.e., repetition suppression for positive primes, and enhancement for negative primes (see Figure 3). It is of note that the large cluster in the left OTC encompasses the canonical coordinates of the VWFA (−40, −50, −14) (Dehaene et al., 2010). The main effects of hemifield and other interactions were all non-significant (*Z* < 2 for all).

**Table 2:** Brain regions showing cross-over interaction between valence and affective priming.

Brain regions	Brodmann area	#voxels	Coordinates			*Z*
			*x*	*y*	*z*	
Left IFG	44/45	2,422	−42	26	18	5.33
	47		−48	44	4	5.10
	45		−48	36	10	4.82
Left ACC	24/32	693	−4	20	52	5.17
Left OTC	37	670	−40	−50	−18	4.76
Left PCC	40/7	470	−38	−58	66	4.30

In subsequent analyses, we examined the effects of congruency separately for positive and negative primes within the four regions showing the valence x congruency interaction (inclusive masking, corrected at *p* < 0.05). When the analysis was restricted to negative primes, the effect of response enhancement was significant at the left IFG (−50, 34, 10, *Z* = 4.86); left ACG (−6, 14, 56, *Z* = 4.86); left OTC (−40, −52, −18, *Z* = 3.60); and left PPC (−34, −50, 50, *Z* = 3/52). On the other hand, positive primes produced significant effects of response suppression in the left IFG (−40, 26, 18, *Z* = 4.12); left ACG (−2, 22, 52, *Z* = 3.22); left OTC (−42, −48, −20, *Z* = 3.53); and left PPC (−44, −54, 64, *Z* = 3/34). Thus, these findings further validate the observed cross-over valence × priming interaction in the left-hemisphere reading network (see Figure 3), which directly reflects the reversal of behavioral priming effects (i.e., facilitatory for positive primes and inhibitory for negative primes).

**Figure 4:**
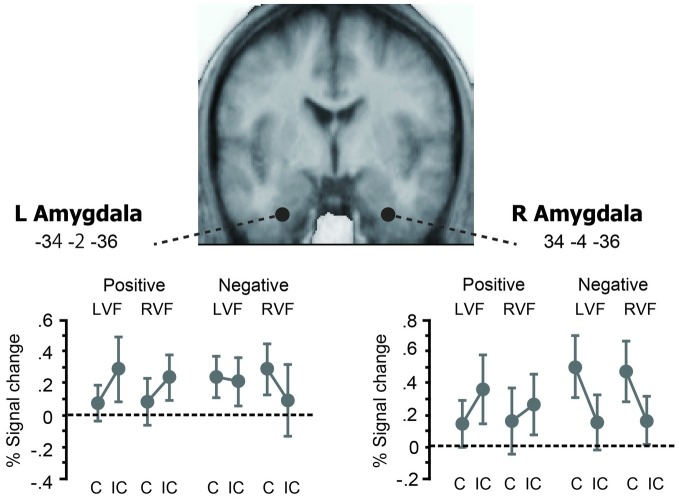
Effects of affective priming in the left and right amygdala ROIs. The left and right amygdala ROIs showed the similar patterns of cross-over interaction between valence and affective priming as those observed in the whole-brain SPM (see Figure 3). The magnitude of this valence × priming interaction did not differ between LVF and RVF, either for the left amygdala or for the right amygdala. Thus, negative and positive primes each showed the same trend of repetition enhancement and repetition suppression as the cerebral reading network, irrespective of the prime hemifield.

Next, we looked at the effects of affective priming in the left and right amygdala ROIs to assess hemispheric differences in early stages of emotion word processing (Figure 4). We first examined the same valence-by-priming interaction as described above for each ROI. This impact of valence on neural priming was significant in the right amygdala (*p* = 0.003, *Z* = 3.43) and showed a non-significant trend in the left amygdala (*p* = 0.08, *Z* = 2.05), suggesting that the amygdala changed its directions of priming in parallel with the left-hemisphere reading network (see Figure 3). On the other hand, the magnitude of priming-by-valence interaction did not differ between LVF primes and RVF primes, either in the left amygdala or in the right amygdala (*p* > 0.05 for both). These findings suggest that amygdala activation is bilateral with slight right hemispheric bias during early stages of emotion word processing.

**Figure 5:**
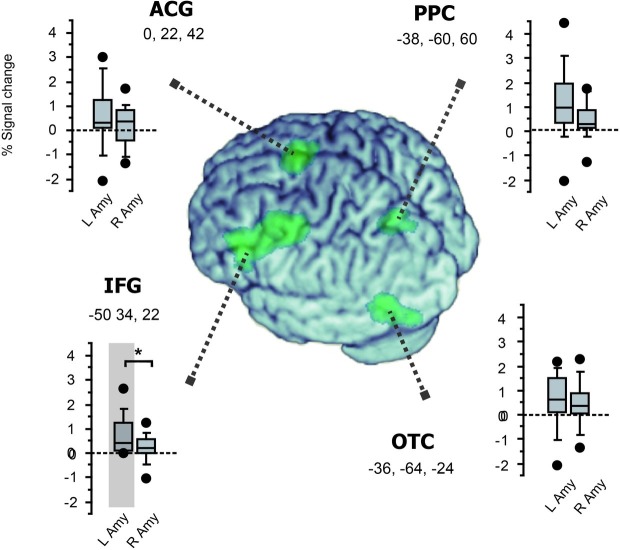
Functional connectivity with the amygdala in the left-hemisphere network involved in fast emotion word processing. Box plots for each region show the magnitude of group-level inter-regional connectivity with the left and right amygdala ROIs. The four cortical regions identified in the whole-brain SPM (see Figure 3) are surface-rendered on the normalized brain using MRIcron (https://www.nitrc.org/projects/mricron). Significant functional coupling was observed only between the left IFG and the left amygdala (shaded in gray, see section “Results”). Moreover, while all the four regions showed the same trend of greater connectivity with the left amygdala than with the right amygdala, this left-vs.-right difference in amygdala connectivity was significant only for the left IFG (^*^p = 0.04).

In PPI analysis, we asked how the bilateral amygdala interacted with the left-hemisphere reading network showing the reversal of priming. Specifically, since the bilateral amygdala exhibited different patterns of priming between positive and negative primes, we examined how this valence x priming interaction was represented in the connection strength between the amygdala and the four cortical components of reading (Figure 5). For the left amygdala, we observed significant increase in functional coupling with the left IFG (*p* = 0.035, *Z* = 3.49) but not with other regions (*p* > 0.4 for ACG and *p* > 0.15 for OTC and PPC, respectively). In contrast, the right amygdala showed no significant change in coupling strength with any of the four regions (*p* > 0.3 for IFG, *p* > 0.3 for ACG, *p* > 0.1 for OTC and *p* > 0.5 for PPC, respectively). For the left IFG, moreover, the left-vs.-right difference in amygdala connectivity was significant (*Z* = 3.16, see Figure 5). Other regions showed no significant left-vs.-right difference in connection strength (*p* > 0.5 for all). Taken together, these findings suggest that the left, but not the right, amygdala exerts a regulatory influence over the reading network *via* the left IFG during emotion word processing.

## Discussion

Behavioral research indicates that emotional stimuli (e.g., fearful faces and scenes) are recognized more rapidly than neutral stimuli (Ohman et al., 2001; Phelps et al., 2006; Bannerman et al., 2009). Interestingly, while reading in itself is not such innate visual behavior but a learned cultural skill, the same behavioral advantage is shown to occur when emotional stimuli are presented as written words (Kousta et al., 2009; Vinson et al., 2014; Yap and Seow, 2014). Emotional word processing is thus likely to involve neurocognitive systems distinct from the classical language network. Specifically, the amygdala complex, known to quickly respond to affective meanings of visual stimuli, is the most likely neural component for reading emotion from words.

Behaviorally, we observed a robust cross-over interaction between valence and priming, whereby affective priming appeared as a facilitatory effect for positive primes and as an inhibitory effect for negative primes, respectively. In fact, emotional valence is shown to change the directions of affective priming, for both verbal and non-verbal stimuli (Hartikainen et al., 2000; Yao and Wang, 2013; Pan et al., 2016). Because masked priming effects generally reflect fast and bottom-up lexico-semantic activation during reading (Forster et al., 2003), our behavioral results suggest that emotional valence of masked primes rapidly changes the functional connectivity in the cerebral reading network.

Indeed, our fMRI results revealed a parallel cross-over interaction at the neural level, which appeared as robust repetition suppression and enhancement in the left frontal and parietotemporal regions. Since these neural structures are known to be closely associated with functional requirements of the task, i.e., the left IFG and OTC for language processing (Price, 2012), visuospatial attention for the PPC (Vidyasagar and Pammer, 2010) and the ACG for cognitive control (Etkin et al., 2015), the observed reversal of neural priming is likely responsible for the robust behavioral effects of facilitatory and inhibitory priming. Our ROI analyses further showed significant valence x priming interaction in the bilateral amygdala (Figure 4), indicating the same trend of suppression and enhancement as the one seen in the left-hemisphere reading network. While the precise neurophysiological basis of repetition suppression and enhancement is an issue of controversy in neuroimaging research (Segaert et al., 2013; Barron et al., 2016; Henson, 2016), the direction of fMRI priming is known to change at different levels of word processing, e.g., lexico-semantic relations (Raposo et al., 2006; Pas et al., 2016), task requirements (Nakamura et al., 2007), and visibility of stimuli (Kouider et al., 2007; Qiao et al., 2010).

For the present study, the observed repetition effects may be attributed to more general and endogenous mechanisms, such as attention and expectation, which play a role in determining the directions of neural priming during visual recognition (Thoma and Henson, 2011; de Gardelle et al., 2013). Given its large effect-size at the behavioral level and broadly distributed effects at the neural level, this reversal of priming may be driven by such global neural mechanism for regulating the entire reading network. Such rapid reconfiguration of functional connectivity can occur since negative stimuli quickly capture attention and strongly engage relevant cognitive resources during visual recognition (Ohman et al., 2001; Bannerman et al., 2009; Lin et al., 2009). Emotional content may also enhance cortical activity at different stages of reading, including visual word-form processing, semantic and contextual analysis (Kissler et al., 2006; Citron et al., 2014).

Our ROI analyses further revealed the similar changes in fMRI priming in the bilateral amygdala. While the amygdala complex has been reported to show different patterns of hemispheric dominance during reading (see section “Introduction”), this particular finding concurs with previous studies showing bilateral amygdala activation during emotion word processing (Eviatar and Zaidel, 1991; Naccache et al., 2005; Nakic et al., 2006). Since masked priming effects reflect fast bottom-up activation during visual word recognition (Dehaene et al., 2006; Kouider and Dehaene, 2007), the present finding suggests that masked emotion words activate the bilateral amygdala outside the classical reading network in early stages of reading.

In PPI analyses, we observed that the valence-by-priming interaction was represented in the connection strength between the left amygdala and the left IFG. By contrast, the right amygdala showed no significant coupling with the left-hemisphere reading network identified in the SPM. Thus, while ROI analyses revealed bilateral effects of affective priming in the amygdala, only the left amygdala produced significant changes in functional connectivity with the left IFG as a function of affective congruency. Collectively, these findings suggest that the left and right amygdala nuclei are both sensitive to affective meanings during the early stage of visual word recognition, whereas only the left amygdala plays a role in regulating the whole reading network. The present results provide direct neuroimaging evidence supporting the proposal that fast amygdala response broadly modulates the neural activity of the language network (Landis, 2006). Such global regulation of task-relevant neural systems may rely exclusively on the left amygdala, which should be linked with other left-hemisphere regions more strongly than its right homologue. Our PPI results suggest that the long-distance control of the reading network is mediated by the emotion regulation circuit linking the amygdala and the inferior frontal cortex.

Specifically, the ventrolateral prefrontal region, which showed the highest effect of priming × valence interaction (see Table 2 and Figure 3), is known to receive inputs from the amygdala and play a role in regulating negative emotion (Quirk and Beer, 2006; Ray and Zald, 2012). This ventrolateral IFG may be also involved in switching the directions of fMRI priming according to the semantic content of visual stimuli (Pas et al., 2016). Moreover, the basal amygdala targeted in the present ROI analyses has dense reciprocal projections *via* the uncinate fasciculus to the orbital part of the IFG (Salzman and Fusi, 2010; Thiebaut de Schotten et al., 2012), i.e., an inferior frontal subregion which showed strong prime-by-valence interaction (see section “Results”). Since the ventrolateral and orbital parts of the IFG also have dense structural and functional connections (Petrides, 2005; Salzman and Fusi, 2010), the observed amygdala-IFG coupling suggests that these emotion circuits play a regulatory role in generating different priming patterns in the left hemisphere. This interpretation seems in good accordance with a recent meta-analysis showing that the orbital IFG is involved in the integration of semantics and emotion (Belyk et al., 2017).

While only the left IFG showed significant change in amygdala connectivity in PPI analyses, it is of note that other components of the reading network could have more constant functional coupling with the amygdala during the semantic judgment task. Given that the amygdala has strong structural connections with the OTC (Catani et al., 2003; Fairhall and Ishai, 2007) and ACG (Etkin et al., 2015), both of these regions may receive sustained inputs from the amygdala during emotion processing. Some caution may be thus needed since the observed IFG-amygdala coupling does not preclude the possible sustained participation of these structures during affective processing.

In an influential neurocognitive model of cultural acquisition, Dehaene and Cohen (2007) propose that visual expertise for written words develops in the ventral visual cortex by reusing occipitotemporal neurons used for recognizing faces in the primate brain. As shown by a recent electrophysiology study by Ponz et al. (2014), emotion word processing may represent an instance of such cultural recycling of pre-existing neural resources, since our fMRI data show that masked emotion words activate the same parts of the bilateral amygdala involved in fearful face processing. If this is the case, such fast neural response may rely not on the occipitotemporal cortex for fine-grained visual analysis but on a subcortical visual pathway linking the pulvinar and the amygdala during fast processing of facial expressions (Garvert et al., 2014; McFadyen et al., 2017; Burra et al., 2019). Indeed, a recent magnetoencephalography study suggests that this subcortical-amygdala route is functioning globally irrespective of spatial frequency and emotion (McFadyen et al., 2017). Semantic content of visual stimuli may be also partially extracted from low-frequency information, because some behavioral studies show significant priming effects from faces (Kouider et al., 2011) and characters (Yeh et al., 2012) in peripheral vision, which has only limited sensitivity to high spatial frequency information. Either way, further studies are needed to determine the potential and limits of the fast subcortical pathway in reading.

In conclusion, while normal reading is generally known to rely on neural components involved in phonological and lexico-semantic activation in the left hemisphere, our fMRI results show that expert reading does not uniquely consist of a well-ordered series of neural processes but involves more distributed and evolutionarily older non-linguistic systems than thought in neurocognitive models of reading. Specifically, we observed that the bilateral amygdala and left orbitofrontal cortex, located outside the classical reading network, constitute tightly interconnected neural components for recognizing the emotional content from written words. Uncovering these supplemental components may help broaden our understanding about the neural basis of normal and impaired reading.

## Data Availability Statement

The datasets generated for this study are available on request to the corresponding author.

## Ethics Statement

The studies involving human participants were reviewed and approved by the ethics committee of Kyoto University Graduate School of Medicine. The patients/participants provided their written informed consent to participate in this study.

## Author Contributions

KN and AU designed the research. KN and TI performed the research, analyzed the data, and wrote the paper.

### Conflict of Interest

The authors declare that the research was conducted in the absence of any commercial or financial relationships that could be construed as a potential conflict of interest.

## Footnotes

^1^http://www.fil.ion.ucl.ac.uk/spm/

^2^http://www.gpower.hhu.de
